# Cognitive function in long-term survivors after treatment for brain metastases compared with normative samples

**DOI:** 10.1093/nop/npaf083

**Published:** 2025-08-04

**Authors:** Guro Lindviksmoen Astrup, Monica Kløvstad Siksjø, Nina Aass, Guido Alves, Kolbjørn Kallesten Brønnick, Marianne Jensen Hjermstad, Stein Kaasa, Astrid Telhaug Karlsson, Nils Inge Landrø, Ole-Bjørn Tysnes, Therese Seierstad, Olav E Yri

**Affiliations:** European Palliative Care Research Centre (PRC), Institute for Clinical Medicine, Oslo, Norway; Department of Oncology, Oslo University Hospital, Oslo, Norway; Institute for Clinical Medicine, University of Oslo, Oslo, Norway; Department of Radiology, Østfold Hospital Trust, Grålum, Norway; European Palliative Care Research Centre (PRC), Institute for Clinical Medicine, Oslo, Norway; Department of Oncology, Oslo University Hospital, Oslo, Norway; Department of Neurology, Stavanger University Hospital, Stavanger, Norway; Department of Chemistry, Bioscience and Environmental Engineering, University of Stavanger, Stavanger, Norway; Centre for Movement Disorders, Stavanger University Hospital, Stavanger, Norway; Department of Social Studies, University of Stavanger, Stavanger, Norway; European Palliative Care Research Centre (PRC), Institute for Clinical Medicine, Oslo, Norway; Department of Oncology, Oslo University Hospital, Oslo, Norway; Department of Oncology, Oslo University Hospital, Oslo, Norway; European Palliative Care Research Centre (PRC), Institute for Clinical Medicine, Oslo, Norway; Department of Oncology, Oslo University Hospital, Oslo, Norway; Clinical Neuroscience Research Group, Department of Psychology, University of Oslo, Oslo, Norway; Department of Neurology, Haukeland University Hospital, Bergen, Norway; Department of Clinical Medicine, University of Bergen, Bergen, Norway; Division for Radiology and Nuclear Medicine, Oslo University Hospital, Oslo, Norway; European Palliative Care Research Centre (PRC), Institute for Clinical Medicine, Oslo, Norway; Department of Oncology, Oslo University Hospital, Oslo, Norway

**Keywords:** brain metastases, cancer survivor, cancer-related cognitive impairment, neurocognitive functioning, radiotherapy

## Abstract

**Background:**

Few studies have examined cognitive function in long-term survivors with brain metastases (BM). The aim of this study was to assess performance-based cognitive function and compare it with normative samples.

**Methods:**

Patients with ≥2-year survival after a first-time BM diagnosis completed neuropsychological tests assessing the following neurocognitive domains: processing speed, executive functioning, mental flexibility, visual learning and memory, verbal learning and memory, attention, and working memory. Test scores were converted into standardized *z*-scores using normative sample means and standard deviations (SD). The proportions of patients with an individual *z*-score of ≤−1 and ≤−1.5 SD below the normative sample means were calculated to indicate subclinical and clinically significant impairment.

**Results:**

Forty-one patients (median age 58, 24 women) completed assessments at a median of 32 months (min–max: 26–47) after the BM diagnosis. On a group level, patients performed poorer than normative samples in tests within all domains except verbal learning and memory. The deviation was most pronounced in a subtest assessing mental flexibility. The proportion of patients with subclinical or clinically significant impairment ranged from 29% to 59% and 7% to 51% between domains, respectively. Seventy-three percent had at least subclinical and 59% had clinically significant impairment in at least 1 domain.

**Conclusions:**

Long-term survivors with BM performed poorer than normative samples within nearly all neurocognitive domains, particularly executive functioning and mental flexibility. This domain is important for daily living and social functioning, and clinicians should provide information about the implications of BM and tailor patient care to optimize functioning and quality of life.

**Key Points**

- Many long-term survivors with brain metastases had impaired cognitive function.
- The executive function and mental flexibility domain was most affected.

**Importance of the Study**

This study provides new information about objective cognitive function in patients with long-term survival after brain metastases (BM). Our findings suggest that they perform poorer than the general population across several neurocognitive domains, and that a substantial proportion of patients suffers from cognitive impairment in at least 1 neurocognitive domain. With increased incidence of BM and longer survival time after diagnosis, more long-term survivors will need assessment of cognitive function. Given the importance of cognitive function in daily living, patients may need follow-up tailored to their specific needs for care to optimize daily living and quality of life.

Patients with brain metastases (BM), especially those treated with radiotherapy (RT) to the brain, experience deficits in cognitive functioning. This primarily affects attention, memory, and executive functions, and can severely impact patients’ and caregivers’ quality of life.^1,2^ BM occurs in around 20%–40% of patients with advanced-stage cancer,^3^ most commonly in those with lung and breast cancer, and melanoma.^4^ Available treatments include surgery, stereotactic radiotherapy (SRT), whole-brain radiotherapy (WBRT), chemotherapy, immunotherapies, and molecularly targeted treatments, either as single or combination treatments. Patients with BM generally have a poor survival prognosis, but the patient population is heterogeneous in terms of disease- and treatment-related factors, and the length of survival after BM diagnosis varies greatly.^5^ Subgroups with more favorable survival prognosis are identified based on age, performance status, extracranial metastases, number of BM, and tumor subtype. These factors are included in the diagnosis-specific Graded Prognostic Assessment index.^6^ Median survival in the most favorable prognostic groups among patients with lung and breast cancer, and melanoma may be as long as 47, 37, and 34 months, respectively.^5^ Although our understanding of biological mechanisms and clinical outcomes in these subgroups is growing, less is known about long-term survivors’ cognitive function.

Cognitive function can be assessed using both patient-reported outcome measures and performance-based neuropsychological tests. Studies using both methods often find absent or weak associations between patient-reported cognitive function and neuropsychological test scores.^7^ Thus, neuropsychological tests are commonly viewed as the gold standard. In studies using neuropsychological tests in patients with BM, change in cognitive performance has been observed across several cognitive constructs, including, but not limited to, verbal learning and memory, executive function, information processing speed, and fine motor coordination.^8^ Cognitive impairment is frequently assessed by comparing test results with normative samples and calculating *z*-scores. Commonly, *z*-scores ≤−1.5 standard deviations (SD) below the normative sample are used to indicate impairment, while some apply ≤−1 SD or a more conservative ≤−2 SD.

Cognitive deterioration has been found to affect approximately 90% of patients after WBRT.^2^ In a meta-analysis of studies assessing objective cognitive function prior to and at least once after WBRT, van Grinsven et al.^8^ found that WBRT is associated with reduced cognitive function in most domains at short-term follow-up (1–4 months), but cognitive function stabilized or returned to baseline in patients assessed at least 9–15 months after WBRT. In patients who received SRT, a decline in cognitive function was sometimes observed shortly after treatment, but the majority of patients returned to baseline scores or showed no decline within a year after treatment.^9^ Whether scores decline, remain stable, or improve beyond the first year is less studied. In a randomized controlled trial of patients with a median follow-up of 24 months, patients treated with SRT experienced less cognitive deterioration than those treated with WBRT (37%–60% and 75%–91%, respectively).^10^ In a small study of 8 patients with a median follow-up of 5.4 years after WBRT, 2 patients reported subjective impairment, and 3 scored below average on neuropsychological tests.^11^ Many trials suffer from methodological limitations such as lack of information about normative data and correction for potential retest effects (ie, improvement in scores due to familiarity with tests). Also, disentangling the cognitive effects of RT from the effects of the disease itself and from the effects of other treatments remains difficult.^12^ Further knowledge of cognitive function in long-term survivors after BM is warranted due to limited knowledge and understanding of the implications for patients. The objective of this study was to assess cognitive function using neuropsychological tests in patients who had survived ≥2 years after a BM diagnosis. Limited evidence from previous research prevented a specific hypothesis, but we hypothesized that patients would perform poorer than normative samples. To evaluate this, we aimed to (1) compare performance-based cognitive function in patients to normative samples and (2) assess the proportion of patients with impaired cognitive function.

## Methods

This prospective study is part of the large, multi-center project «Brain Metastases in Norway—Improved Classification and Treatment» entailing several sub-studies conducted in the central and southeast health regions of Norway between 2017 and 2021. One of these sub-studies^13^ included 912 patients who had newly verified first-time BM from solid cancers, were ≥18 years of age, and were able to provide written informed consent. We identified 164 patients (18%) who were included in the study and who were alive ≥2 years after BM diagnosis and thereby eligible to participate. There were no other exclusion criteria. Compared to those who were not eligible (survival <2 years or died before invitation to the follow-up study), eligible patients were significantly younger (62 vs. 68 years), had more favorable diagnostic characteristics (ie, ECOG 0-1, <5 BM, no extracranial metastases, targetable mutation(s) [ie, ALK, EGFR, BRAF, HER-2, KRAS, MSI, NRAS, PD-L1, Progesterone, ROS1, Estrogen]), and more patients were treated with surgery or SRT compared to WBRT as their initial treatment (all *P* < .001). Eighty-nine patients living within a travel distance that allowed for an outpatient visit to the hospital were invited to participate in this follow-up study via letter. Those who accepted were asked to attend a 1-day visit to Oslo University Hospital. The visit included collection of blood samples, an ^18^F-fluoro-deoxy-glucose positron emission tomography/computed tomography (PET/CT) scan of the brain, a cognitive assessment including several neuropsychological tests, and a clinical examination with an oncologist. In this paper, we report on the results from the neuropsychological tests.

### Neuropsychological Tests

A battery of neuropsychological tests was administered. The tests are widely used, standardized psychometric instruments for assessing neurocognitive deficits and cover the main dimensions of higher cognitive functions, like speed, attention, working memory, learning, and recall as well as executive functions. The various subcomponents are also subserved by distinct neural networks^14^ (Table 1).

**Table 1.** Neuropsychological Tests Used in the Study

Neurocognitive domain	Test	Unit of measure	High score indicate
Processing speed	SCWT Words and SCWT Colors	Number of correctly read or named colors (range)	Good performance
	TMT A	Completion time (seconds)	Poor performance
Executive functioning and mental flexibility	SCWT Colored words	Number of correctly named colors (range)	Good performance
	TMT B	Completion time (seconds)	Poor performance
Visual learning and memory	CVMT	Total number of hits, false alarms and delayed recognition (range)	Good performance
Verbal learning and memory	CVLT-II	Total number of recalled items (range)	Good performance
Attention and working memory	CVLT-II List A trial 1	Total number of recalled items (range)	Good performance
	WAIS IV Digit span	Total number of correctly repeated sequences (range)	Good performance

Abbreviations: CVLT-II = California Verbal Learning Test II; CVMT = Continuous Visual Memory Test; SCWT = Stroop Color and Word Test; TMT = Trail Making Test; WAIS = Wechsler Adult Intelligence Scale.

Processing speed and executive functioning were evaluated with the Stroop Color and Word Test (SCWT)^15,16^ and the Trail Making Test (TMT).^17^ The SCWT measures the ability to suppress a habitual response to a stimulus in favor of an unusual one (speed, selective attention, and inhibitory control). The test consists of 3 parts, each with 100 words in black or colored ink (red, green, or blue). Patients were instructed to read color names in black ink (part 1), name the ink color of “*xxxx*” (part 2), and name the incongruent ink color of the color names (part 3). The score for each part is the number of correctly read or named colors within 45 seconds. The TMT, where patients were asked to draw a line between connecting numbers (part A) and sequentially between connecting numbers and letters (part B), measures processing speed and mental flexibility. The score is the amount of time (seconds) spent completing each task.

Visual learning and memory were evaluated with the Continuous Visual Memory Test (CVMT)^18^ that consists of 112 figures, of which 7 are target figures. Patients were asked to indicate figures they had seen before, and the number of figures identified, and false positives were scored as a total score and a d-prime score (ie, transformed score based on hit rate and false alarm rate). In a delayed recognition trial performed after 30 min, patients identified the target figures.

Verbal learning and memory were evaluated with the California Verbal Learning Test (CVLT-II),^19^ where patients were asked to repeat a 16-word list 5 times (List A), scored as total number of correct items and learning slope (ie, mean number of new words learnt between subsequent repetitions). Short-term delay and long-term delay (ie, after 20 min) retests with free recall were also performed.

Attention and working memory were evaluated with the Wechsler Adult Intelligence Scale (WAIS) IV Digit span,^20^ where patients were asked to repeat increasingly longer strings of random numbers, first forward and then backward by reversing the sequence. In the last task, patients were asked to repeat random numbers in ascending order. The test continued until they failed twice or reached 16 completed sequences, and the total score is the combined scores of the 3 tasks. The results from trial 1 from List A in the CVLT-II were also used to assess attention.^18^

### Statistical Analyses

Descriptive statistics and frequency distributions were used to present demographic and clinical data. Differences in demographic and clinical characteristics between patients who were included in the long-term follow-up and patients who were not were tested using the *χ*^2^ test for categorical data and independent samples *t*-tests for (normally distributed) continuous data. Analyses were performed using Stata version 18 (StataCorp, College Station, Texas). Tests were 2-tailed, and associations were considered significant if *P* < .05.

Each neuropsychological subtest was scored according to standardized scoring criteria. Then, individual test scores of patients were transformed into equivalent *T*-scores where appropriate and converted into standardized *z*-scores with the use of means and SD of normative samples from published norm data in manuals and articles, as well as a control group from the Norwegian ParkWest Study^21^ (for the SCWT, see Supplementary Material 1). Individual test scores were matched for age, sex, and level of education where appropriate: scores for the CVMT and WAIS IV Digit span were matched for age; scores for the CVLT-II and SCWT were matched for age and sex; and scores for the TMT were matched for age and education (for patients above 55 years). Lower *z*-scores represent worse performance, including *z*-scores for the TMT that have been reversed to be included in the overall neurocognitive domain scores. Overall scores were calculated using the mean of the *z*-scores of the available tests within a domain. Group-level differences in *z*-scores between patients and normative samples for each test and each domain were evaluated using Cohen’s criteria for small, medium, and large effects (ie, Cohen’s d 0.2, 0.5, and 0.8).

For each test, we calculated the proportion of patients with an individual *z*-score of ≤−1 SD and ≤−1.5 SD below the mean of the normative samples, considered as 2 levels of cognitive impairment: at least subclinical and clinically significant impairment.^14^ Next, at least subclinical and clinically significant impairment in each neurocognitive domain was defined as a *z*-score of ≤−1 SD and ≤−1.5 SD below the normative mean in at least 50% of the test scores (ie, 1 test in domains containing 2 subtests; 2 tests in domains containing 3 or 4 subtests; 3 tests in domains containing 5 subtests).

## Results

### Patient Characteristics

**Figure 1.**
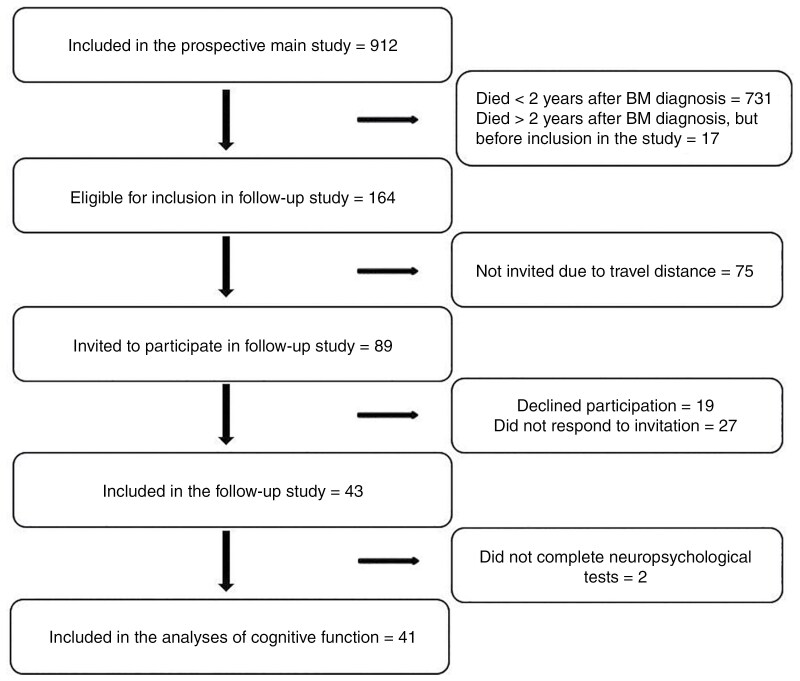
Flow chart of patient recruitment. Abbreviation: BM = brain metastases.

Patient recruitment and compliance are outlined in Figure 1. There were no significant differences in age, sex, or any clinical characteristics (ie, ECOG, time since primary diagnosis, presence of extracranial metastases or targetable mutations, number of BM, primary BM treatment) between those who were invited to participate in the follow-up study (*n* = 89) and those who were not due to travel distance (*n* = 75). Of the 89 invited patients, 43 (48%) accepted the invitation and were included in the study. Forty-one of these patients completed the neuropsychological tests and are included in the analyses. There were no significant differences between those who accepted the invitation (*n* = 43) and those who did not (*n* = 46) in any of the aforementioned patient characteristics.

The patients were included at a median of 32 months (min–max: 26–47) after inclusion in the main study. Demographic and clinical characteristics of the participants are presented in Table 2. The median age at the time of inclusion in the follow-up study was 61 (min-max: 31-83) and 59% were women. Most had a primary diagnosis of lung cancer (46%), breast cancer (22%) or melanoma (22%). Sixty-one percent had 1 BM at the time of BM diagnosis; the most common location was the right hemisphere. Initial BM treatment was surgery (32%), SRT (44%), WBRT (17%) or systemic treatment (7%) (see Supplementary Material 2). Of the 10 patients who underwent surgery and postoperative SRT as initial treatment, 2 later received WBRT for intracranial progression. Of the 18 patients who received SRT as initial treatment, 3 later received SRT and 2 received SRT and later WBRT for intracranial progression. Of the 3 patients who received systemic treatment as initial treatment, one later received SRT. Thus, a total of 11 patients received WBRT at any point before inclusion in this follow-up study. WBRT was given without hippocampal avoidance.

**Table 2.** Demographic and Clinical Characteristics of the Participants at the Time of Diagnosis of BM (*n* = 41)

Characteristic		*N* (%)
Age, *median*, years (min–max)	58 (28–81)	
Sex	Female	24 (59)
	Male	17 (41)
Education	Primary	9 (22)
	Secondary	14 (34)
	College/University	16 (39)
	Unknown	2 (5)
ECOG performance status at BM diagnosis	0	15 (37)
	1	17 (41)
	2	8 (20)
	3–4	0 (0)
	Unknown	1 (2)
Primary tumor	Lung	19 (46)
	Melanoma	9 (22)
	Breast	9 (22)
	Other	4 (10)
Histology	Adenocarcinoma	19 (46)
	Squamous cell carcinoma	4 (10)
	Melanoma	7 (17)
	Other	9 (22)
	Unknown	2 (5)
Targetable mutation^a^	Yes	30 (73)
Extracranial metastases	Yes	28 (68)
Number of BM at BM diagnosis	1	25 (61)
	2–4	6 (15)
	≥5	10 (24)
Location of BM^b^	Left hemisphere	16 (39)
	Right hemisphere	18 (44)
	Frontal lobe	12 (29)
	Parietal lobe	7 (17)
	Temporal lobe	6 (15)
	Occipital lobe	8 (20)
	Cerebellum	15 (37)
	Thalamus	1 (2)
	Brain stem	1 (2)
Initial treatment for BM	Surgery only	3 (7)
	Surgery and postoperative SRT	10 (24)
	SRT	18 (44)
	WBRT	7 (17)
	Systemic treatment	3 (7)
Subsequent RT for BM^c^	None	25 (61)
	SRT	12 (29)
	SRT and WBRT	4 (10)
Subsequent RT other	Bone, lung, lymph node	14 (34)
Subsequent systemic cancer treatment	None	7 (17)
	Chemotherapy	15 (37)
	Immunotherapy	22 (54)
	Targeted therapy	12 (29)
	Endocrine therapy	5 (12)

^a^Mutation or positivity for at least one of the following: ALK, EGFR, BRAF, HER-2, KRAS, MSI, NRAS, PD-L1, Progesterone, ROS1, Estrogen.

^b^Patients may have lesions in several locations of the brain, and percentages do not add up to 100.

^c^Of the 10 patients who underwent surgery and postoperative SRT as initial treatment, 2 later received WBRT for intracranial progression. Of the 18 patients who received SRT as initial treatment, 3 later received SRT and 2 received SRT and later WBRT for intracranial progression. Of the 3 patients who received systemic treatment as initial treatment, one later received SRT.

Abbreviations: BM = brain metastases; ECOG = Eastern Cooperative Oncology Group; RT = radiotherapy; SRT = stereotactic radiotherapy; WBRT = whole-brain radiotherapy.

### Cognitive Function ≥2 Years After Diagnosis of BM

#### Group level.

**Figure 2.**
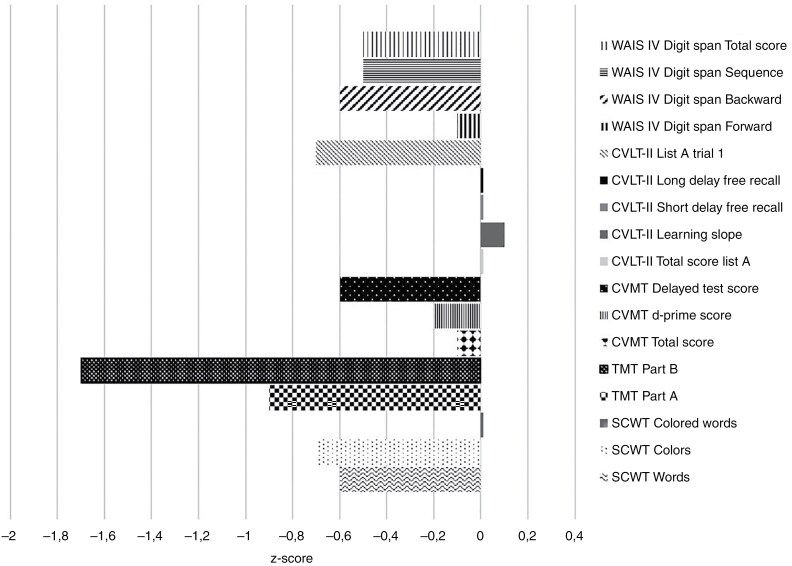
Neuropsychological test scores for patients compared to normative samples (*z*-scores). Abbreviations: CVLT = California Verbal Learning Test; CVMT = Continuous Visual Memory Test; MOS-Cog = Medical Outcomes Study Cognitive Functioning Scale; SCWT = Stroop Color and Word Test; TMT = Trail Making Test; WAIS = Wechsler Adult Intelligence Scale.

—Neuropsychological test scores and comparison with appropriate normative samples are summarized in Table 3 and Figure 2. There were differences with medium and large size effects using Cohen’s d between patients and normative samples on subtests within several domains. The effect sizes were small across subtests in the verbal learning and memory domain (0.00–0.09), and medium size within the processing speed domain (0.54–0.65). There was more variation in effect sizes across subtests in the other domains, ranging from 0.10 to 0.64 for visual learning and memory, 0.14 to 0.65 for attention and working memory, while 0.01 and 1.31 for the 2 subtests in the executive functioning and mental flexibility domain.

**Table 3.** Neuropsychological Test Scores for Patients Compared to Normative Samples

Neurocognitive domain	Test		Patients				Matched to normative samples		
			*n*	Mean	SD	Min–max	Mean *z*-score	SD	Cohen’s *d*
Processing speed	SCWT	Words	41	77.0	20.3	38–131	−0.6	1.7	0.54
		Colors	37	56.5	14.6	26–90	−0.7	1.5	0.65
	TMT	A	40	48.2	37.5	15–204	−0.9	2.4	0.65
Executive functioning, mental flexibility	SCWT	Colored words	38	32.3	14.7	2–70	0.0	1.8	0.01
	TMT	B	41	121.7	70.7	40–343	−1.7	2.3	1.31
Visual learning and memory	CVMT	Total score	41	73.3	8.1	51–93	−0.1	1.4	0.10
		d-prime score	41	1.7	0.6	0.4–3.8	−0.2	1.3	0.23
		Delayed test score	41	3.0	1.6	0–7	−0.6	1.1	0.64
Verbal learning and memory	CVLT-II	Total score list A	40	44.4	14.6	14–75	0.0	1.4	0.00
		Learning slope	40	1.4	0.6	−0.1 to 2.7	0.1	1.1	0.09
		Short delay free recall	40	9.3	4.2	1–16	0.0	1.2	0.02
		Long delay free recall	41	9.7	4.6	0–16	0.0	1.3	0.05
Attention and working memory	CVLT-II	List A trial 1	41	5.2	2.2	2–12	−0.7	1.3	0.65
	WAIS IV Digit span	Forward	41	8.5	1.8	5–13	−0.1	1.0	0.14
		Backward	41	6.6	1.7	3–10	−0.6	0.8	0.61
		Sequence	41	6.6	1.9	3–12	−0.5	0.8	0.55
		Total score	41	21.7	4.4	15–35	−0.5	0.7	0.51

Abbreviations: CVLT = California Verbal Learning Test; CVMT = Continuous Visual Memory Test; SD = standard deviations; SCWT = Stroop Color and Word Test; TMT = Trail Making Test; WAIS = Wechsler Adult Intelligence Scale.

*Note*: *z*-scores for TMT are reversed so negative scores reflect poorer scores than the normative population. Cohen’s *d* = Cohen’s criteria for small (0.2), medium (0.5), and large (0.8) effects.

#### Individual level.

—The percentage of patients with at least subclinical impairment (≤−1 SD) in each domain ranged from 29% in the attention and working memory, verbal learning and memory, and visual learning and memory domains to 41% in the processing speed domain, and 59% in the executive functioning and mental flexibility domain (Table 4). Seven percent of the patients exhibited clinically significant impairment (≤1.5 SD) in the attention and working memory domain, 12% in both the visual learning and memory domain and in the verbal learning and memory domain, 24% in the processing speed domain, and 51% in the executive functioning and mental flexibility domain. Seventy-three percent had at least subclinical impairment and 59% had clinically significant impairment in at least 1 neurocognitive domain. There was a large variation in number of domains with impairment in the study population: 1 domain (15% at least subclinical, 24% clinically significant), 2 domains (24% at least subclinical, 29% clinically significant), 3 domains (15% at least subclinical, 5% clinically significant), 4 domains (17% at least subclinical, 0% clinically significant), and all domains (1 patient). Among patients with impairment in only 1 domain, none had impairment in processing speed or attention and working memory. Among those with impairment in 2 or 3 domains, executive function and mental flexibility were the most commonly affected domains, followed by processing speed.

**Table 4.** Percentage of Patients with Subclinical or Clinically Significant Impairment in Each Domain (*n* = 41)

Neurocognitive domain	Subclinical impairment (*z* ≤−1 SD)	Clinically significant impairment (*z* ≤−1.5 SD)
	*n* (%)	*n* (%)
Processing speed	17 (41%)	10 (24%)
Executive functioning and mental flexibility	24 (59%)	21 (51%)
Visual learning and memory	12 (29%)	5 (12%)
Verbal learning and memory	12 (29%)	5 (12%)
Attention and working memory	12 (29%)	3 (7%)

Abbreviation: SD = standard deviations.

## Discussion

Performance-based cognitive function was assessed in 41 patients surviving more than 2 years after first-time BM diagnosis. Compared to normative samples, the patients performed worse in all domains of cognitive function, except for verbal learning and memory. There were medium effect sizes on subtests reflecting processing speed, attention, working memory, and visual memory. A large effect size on a subtest assessing mental flexibility emerged, a main dimension of executive functioning. Furthermore, 51% of patients showed clinically significant impairment in the executive functioning and mental flexibility domain, whereas only 7%–24% scored outside the normal range in the other domains. Patients performed very differently for the 2 subtests measuring executive functioning and mental flexibility (ie, SCWT Colored words and TMT-B). While both tests assess aspects of executive functioning, they primarily evaluate distinct cognitive processes: the SCWT Colored words test predominantly measures inhibitory control, whereas the TMT-B primarily assesses cognitive flexibility and set-shifting abilities.^22^ Impaired executive functions can have a major impact on everyday life; like planning, performing, and switching between activities, functions important for both work life and daily life, as well as social functioning. Dysfunctional inhibitory control can also influence the ability to regulate negative emotions.^14^

On the other hand, normal verbal learning and memory is a positive message to this patient group given that it affects all areas of daily life that includes verbal information. Other studies have reported impairment in verbal memory as well as other domains in patients after BM diagnosis. Studies assessing cognitive function *prior to* BM treatment have reported somewhat conflicting results. Van Grinsven et al.^23^ evaluated patients with newly diagnosed BM referred to RT and found that 69% reported a subjective decline in cognitive performance when compared to their premorbid (ie, precancer) level, most often described as attention, memory, and thinking. Furthermore, they found that almost 80% had objective deficits in at least 1 domain prior to the start of RT, most commonly in memory. Patients performed better than the normative population in tests for attention and visuospatial functioning prior to RT, poorer for psychomotor speed and processing speed, while it varied for different memory tests. A potential explanation for this may be that attention and memory are affected more by BM treatment than the BM itself.^24^ Worth noting is that verbal and visual memory were combined in 1 domain in their study, although verbal and visual memory may be affected by processes in different hemispheres. In contrast, Habets et al.^25^ found that patients performed poorer than healthy controls for verbal memory, attention, working memory, executive functioning, and visuoconstruction prior to SRT. They also found a slightly smaller percentage (53%) with impairment in at least 1 neurocognitive domain compared to van Grinsven et al., most frequent in verbal memory.

Results are more consistent between studies assessing long-term cognitive function *after* BM treatment. In their follow-up study, van Grinsven et al.^26^ found that while 50% reported subjective decline in at least 1 cognitive domain, cognitive function across all domains declined both at 3 and ≥11 months post-RT, most frequent for memory and processing speed. Another study of patients surviving at least 12 months also found that a number of patients experienced cognitive deterioration in all domains at 3 months, most often in memory and recognition.^9^ Most patients treated with a combination of SRT and WBRT experienced cognitive impairment at both 3 and 12 months (both 94%), compared to those treated with SRT alone, although the incidence of cognitive decline increased from 3 to 12 months in the SRT group (46% and 60%).^9^ Although memory was decreased in both studies, a stabilization or slight improvement was seen around 12 months after BM diagnosis, which may explain why memory was less affected in our patients after >24 months. Several methodological factors can influence the varying results. Previous studies as well as ours are small and include patients with different demographic and clinical characteristics. Also, different neuropsychological tests are used, and the neuropsychological tests are classified under different domains. Lastly, different approaches are used to identify patients with impaired function. For example, van Grinsven et al.^23,26^ defined impairment in each domain as *z*-score ≤−1.5 on any of the administered tests within the domain, while we defined impairment as ≤−1 and ≤−1.5, respectively, in at least 50% of the tests within the domain.

### Levels of Cognitive Impairment

More patients experienced at least subclinical impairment compared to clinically significant impairment in cognitive function. Most previous studies have used *z*-scores ≤−1.5 SD below the normative sample to indicate impairment, while some apply ≤−1 SD or a more conservative ≤−2 SD. Neuropsychological tests were initially developed to identify more significant impairments, such as those associated with neurodegenerative diseases and brain injuries. It is possible that measurement error may obscure at least some of the subtle cognitive decline.

The normative datasets we used for analysis do not explicitly list mild cognitive impairment as an exclusion criterion. Cognitive decline is strongly correlated to age, which makes it difficult to differentiate cognitive impairment from normal reduction in cognitive abilities due to aging. It is fair to assume that normative datasets based on individuals screened by only a clinical assessment, includes at least some elderly participants with a subtle, but progressive cognitive decline. This bias reduces the sensitivity to detect accurately the milder presentations of cognitive impairment since the normative dataset itself might be based at least partially on elderly individuals with mild cognitive impairment.^27^ For example, Stricker et al.^28^ found an unusually low prevalence of low test scores (≤−1 SD) on an auditory verbal learning test when applying the original version of Mayo’s Older Americans Normative Studies dataset to an unimpaired sample. They hypothesized that the potential inclusion of mildly cognitively impaired individuals in the normative data led to reduced sensitivity. This stands in stark contrast to our findings, where we observed a substantially higher prevalence of low test scores across all administered tests. This skewness indicates more widespread cognitive decline in our sample compared to what would be expected in a normative population. Additionally, discrepancies may arise from misunderstandings regarding the specific neurocognitive domains affected. Ahles et al.^29^ found that many cancer survivors reported memory deficits when in fact the underlying issue was related to attention impairment. These patients performed within the normal range on neuropsychological tests measuring verbal memory, but not on the attention subtest. This observation is consistent with our findings from the CVLT-II verbal learning and memory test. A hypothesis is that patients perceived memory deficits stem from difficulties with encoding information, leading to inefficient storage and reduced availability for later recall.^30^ This could indicate a difference between memory impairment attributed to cancer treatment and the one commonly found with debilitating neurodegenerative diseases, such as dementia. Some forms of cognitive rehabilitation aimed to manage/compensate for cognitive deficits in cancer patients have shown promising results.^29^

### Factors Associated with Reduced Cognitive Function

In addition to the cancer disease itself as well as RT, several other treatments, including chemotherapy, endocrine therapy, and targeted therapy, have also been associated with cognitive impairment.^31^ Although most patients received some form of RT for BM, our sample was quite heterogeneous in terms of disease and treatment characteristics and too small to perform statistical analyses on differences in characteristics between patients with and without cognitive impairment. Furthermore, previous studies have found that neither demographic characteristics, stress, nor clinical characteristics such as primary tumor, synchronous diagnosis of BM, number of BM, presence of active systemic disease, or use of antiepileptic or sedating drugs were associated with cognitive function.^23,25,26,32^ However, other factors not related to the cancer disease, such as aging, comorbidities, inflammation,^31^ and depression^33^ may impact cognitive function. Clinicians may consider screening for cognitive impairment and potentially reversible factors that may contribute. Potential treatments or coping strategies such as physical activity, mindfulness, and cognitive rehabilitation approaches have shown promising results in other cancer populations^31^ and may be explored in future research.

### Strengths and Limitations

A strength of this study is the evaluation of long-term survivors with substantially longer survival times after BM diagnosis than most previous studies, commonly focusing on patients 3 to 12 months after BM diagnosis. Also, the study included participants from a large, population-based study of unselected patients, reflecting the BM population. Another strength is the comprehensive battery of objective, validated neuropsychological tests covering the most central neurocognitive domains. Limitations are the high number of patients who were not eligible to participate or declined, the small sample size hindering subgroup comparisons, the lack of premorbid or pretreatment assessments of cognitive function, and the lack of assessment of depression. Furthermore, as this study did not include a locally sampled, healthy control group, demographic differences between the included patient group and the normative samples cannot be ruled out. Additionally, different normative samples were used for each test, with variations in corrections for demographic variables. There may also be limitations related to the selection criteria of participants in the normative datasets (see Supplementary Material 1).

## Conclusions

Our findings suggest that a substantial proportion of patients suffer from cognitive impairment in at least 1 neurocognitive domain, most commonly in the executive functioning and mental flexibility domain. Still, our findings also indicate that many patients score within the normal range and probably function normal or close to normal. There is a need for further research on the variation between subclinical and clinically significant cognitive impairment and the implications for functioning. The increasing incidence of BM and length of survival after treatment underscores the need to improve our understanding of the impact of cognitive impairment on activities of daily living and ability to work or maintain personal relationships for both patients and their caregivers. Patients who survive beyond 1–2 years may have entirely different needs for follow-up by oncologists, primary health care teams, or others. Patients and caregivers need timely and adequate information to be able to manage and adapt to alterations in daily life and quality of life. Clinicians need sufficient information to be able to tailor treatment and follow-up to optimize patient-centered care.

## Supplementary material

Supplementary material is available online at *Neuro-Oncology Practice* (https://academic.oup.com/nop/).

npaf083_Supplementary_Materials

## Data Availability

Data will be made available upon reasonable request.
