# Supplementary material for: Cognitive function in long-term survivors after treatment for brain metastases compared with normative samples
Source: Neurooncol Pract. 2025 Aug 4;13(1):178–88. doi: 10.1093/nop/npaf083 (PMC12965655; doi:10.1093/nop/npaf083)
Supplement: npaf083_Supplementary_Materials [file npaf083_supplementary_materials.docx]

**Supplementary material 1 Normative data**

We present here a short overview of the normative data utilized in the analysis of the neurocognitive test results. The tests are organized in alphabetical order.

For our patient group, T-scores and scaled scores calculated from normative data have been converted to z-scores. Additionally, z-scores were computed directly from the normative data presented as standard scores or derived from group means and standard deviations (SD).

*California Verbal Learning Test II:*

Normative data for the California Verbal Learning Test II (CVLT-II) was obtained from the English manual published in 2000 ^1^. The manual contains tables presenting normative data adjusted for sex and seven age groups. Total Score is presented as T-scores in the tables, while Learning Slope, Short Delay Free Recall, Long Delay Free Recall, and List A Trial 1 are presented as standard scores. The normative data were gathered from a sample of 1,087 healthy Americans (522 men, 565 women) between 16 and 89 years of age.

*Continuous Visual Memory Test:*

We utilized two different sources of normative data for the analysis of the Continuous Visual Memory Test (CVMT). The original normative data, as documented in the CVMT manual published in 1988, includes age-adjusted tables for Total Score, d-Prime, and Delayed Recognition ^2^. This data is derived from an American sample of 310 healthy individuals (140 men, 170 women) aged 18 to 91, divided into four age groups. This normative data was used to evaluate performance on the Total Score and d-Prime metrics for patients aged 59 years and younger, as well as to assess the Delayed Recognition score for all age groups. For individuals aged 60 years and older, we used the normative data published by Paolo *et al*. in 1998 to analyze Total Score and d-Prime ^3^. Both publications presented normative data as group mean scores and SD.

The CVMT is sensitive to the cognitive effects associated with aging. In their study, Paolo *et al*. included 177 healthy Americans (74 men, 103 women) from 60 to 94 years old in a study to investigate the reliability of the CVMT normative data for an elderly population. They found that 8 to 50% of the study participants fell below the cutoff scores recommended in the manual for Total Score, d-Prime, and Delayed Recognition. Significant age effects were observed for Total Score and d-Prime, leading to the publication of updated normative data for these two metrics.

*Stroop Color and Word Test:*

Normative data for the Stroop Color and Word Test (SCWT) were derived from a Norwegian sample of 195 adults (103 men, 92 women) aged 39 to 85 years. The participants were part of the healthy control group in the ParkWest Study published in 2009 ^4^. Each participant completed the SCWT as part of a comprehensive neurocognitive test battery. The data were analyzed using multiple regression analysis for the Words, Colors and Colored Words scores. In this analysis, age was treated as a continuous variable, while education level (categorized as primary, secondary, or higher) and sex were included as categorical variables. A backwards stepwise elimination process was employed to reduce the model by removing the least significant predictor if the two-tailed p-value exceeded 0.05. It was determined that education did not have a significant impact on any of the three scores. The findings were adjusted for sex (for Words and Colors) and separated into five distinct age groups with calculated normative mean scores and SD for each group.

*Trail Making Test:*

Normative data for the Trail Making Test (TMT) was obtained from the third revision of the Norwegian manual (TMT-NR3) ^5^. This manual includes tables for tests A and B that are adjusted for age and education (0-12 years and 12+ years) for individuals aged 55 and older. The normative data is presented as the mean time used to complete each test, along with the SD for each group. The data is derived from a study conducted by Tombaugh in 2004 ^6^, which included a sample of 911 healthy Canadians (408 men, 503 women) between 18 and 89 years of age.

*Wechsler Adult Intelligence Scale IV Digit Span:*

Normative data for the Wechsler Adult Intelligence Scale IV (WAIS-IV) Digit Span has been derived from the Norwegian version of the manual published in 2010 ^7^. This manual provides tables for normative data separated into thirteen age groups. The normative data is presented as scaled scores, with a mean of 10 and a standard deviation of 3 for the Total Score, as well as for the three process scores: Forward, Backward, and Sequencing. The normative data were obtained from a sample of 726 healthy Scandinavians (362 men, 364 women) ranging in age from 16 to 74 years. For individuals aged 75 to 90 years, the tables in the Norwegian manual utilize normative data sourced from the American edition of the manual.

Limitations and considerations

It is reasonable to assume some degree of population-level changes over time, such as the Flynn effect (a drift in average IQ scores across decades) ^8^, as well as changes in sociopolitical factors, educational system, lifestyle, culture, and technology, among others ^9^. Caution is particularly warranted when interpreting results from the CVMT, which relies on normative data from 1988 and 1998. Similar considerations apply to CVLT-II and TMT test scores, as the used normative data are more than 20 years old.

These differences are also applicable when comparing samples from different geographical regions, as there may be demographic variations between the source populations from which the samples are drawn. The influence of demographic parameters such as age, sex, and education can vary not only among different tests but also within and between populations ^9^. Most of the test scores (CVLT-II, CVMT, TMT, and WAIS-IV Digit Span for patients older than 74) were compared to normative standards derived from a North American population, while test scores for SCWT and WAIS-IV Digit Span for patients up to 74 years of age were compared to Norwegian or Scandinavian populations, respectively. Only two of the norms (CVLT-II and SCWT) adjust for sex, and only one (TMT) partially adjusts for education.

Another consideration is the lack of explicit exclusion of elderly individuals with mild cognitive impairment (MCI) in the normative datasets. Although thorough neurocognitive assessments were carried out to exclude participants with more obvious cognitive impairment ^1-7^, there is no guarantee that individuals with MCI or subtle cognitive changes were excluded. It is therefore necessary to consider the potential skewness of normative data for older age groups, which could result in falsely higher z-scores for our elderly patients. This bias may lead to an underestimation of cognitive decline in older individuals when compared to these norms, potentially overlooking subtle cognitive changes.

The SCWT normative data is divided into five age groups, from 39 to 85 years old. For patients younger than 39 years, z-scores were calculated by comparing the patient’s score with the youngest age group. This approach is more likely to result in false negatives than false positives, as performance on the SCWT subtests typically decreases with age.

For the CVLT-II, we employed the Norwegian version of the test, ensuring that language differences did not affect the results for this particular assessment.

References Supplementary Material 1

1. Delis DC, Kramer JH, Ober K, Ober BA (2000). CVLT-II California Verbal Learning Test.
2. Trahan DE, Larrabee GJ (1988). Continuous Visual Memory Test: Professional manual. Psychological Assessment Resources.
3. Paolo AM, Tröster AI, Ryan JJ (1998). Continuous Visual Memory Test Performance in Healthy Persons 60 to 94 Years of Age. Arch Clin Neuropsychol 13(4):333-7.
4. Aarsland D, Brønnick K, Larsen JP, Tysnes OB, Alves G; Norwegian ParkWest Study Group (2009). Cognitive impairment in incident, untreated Parkinson disease: the Norwegian ParkWest study. Neurology 72(13):1121-6.
5. Strobel C, Johansen H, Aga O, Bekkhus-Wetterberg P, Brierley M, Egeland J, Follesø K, Rike P-O, Schanke A-K (2018). Manual norsk revidert Trail Making Test (TMT-NR3).
6. Tombaugh TN (2004). Trail Making Test A and B: Normative data stratified by age and education. Arch Clin Neuropsychol 19(2):203-14.
7. Wechsler D (2010). WAIS-IV Wechsler Adult Intelligence Scale - Fourth edition. Manual Del 1 Norsk versjon.
8. Grégoire J, Weiss LG. The Flynn Effect and Its Clinical Implications (2019). In L. G. Weiss (Ed.), WISC-V: Clinical Use and Interpretation (2nd ed). Academic Press 245-270.
9. Karstens AJ, Christianson TJ, Lundt ES, Machulda MM, Mielke MM, Fields JA, et al (2024). Mayo normative studies: regression-based normative data for ages 30-91 years with a focus on the Boston Naming Test, Trail Making Test and Category Fluency. J Int Neuropsychol Soc 30(4):389-401.

**Supplementary material 2 Primary and subsequent treatment for brain metastases**


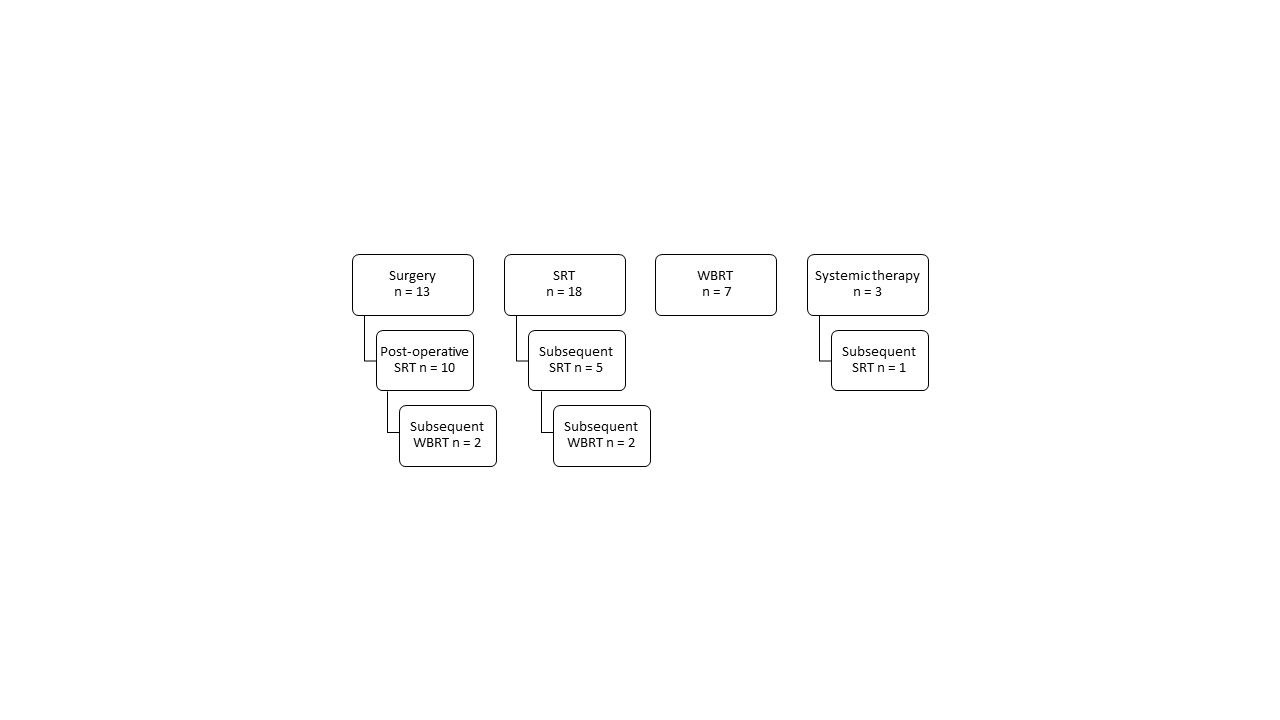


Abbreviations: BM = brain metastases; SRT = stereotactic radiotherapy; WBRT = whole-brain radiotherapy
